# Supplementary figures and images for: Seroprevalence of fourteen human polyomaviruses determined in blood donors
Source: PLoS One. 2018 Oct 23;13(10):e0206273. doi: 10.1371/journal.pone.0206273 (PMC6198985; doi:10.1371/journal.pone.0206273)

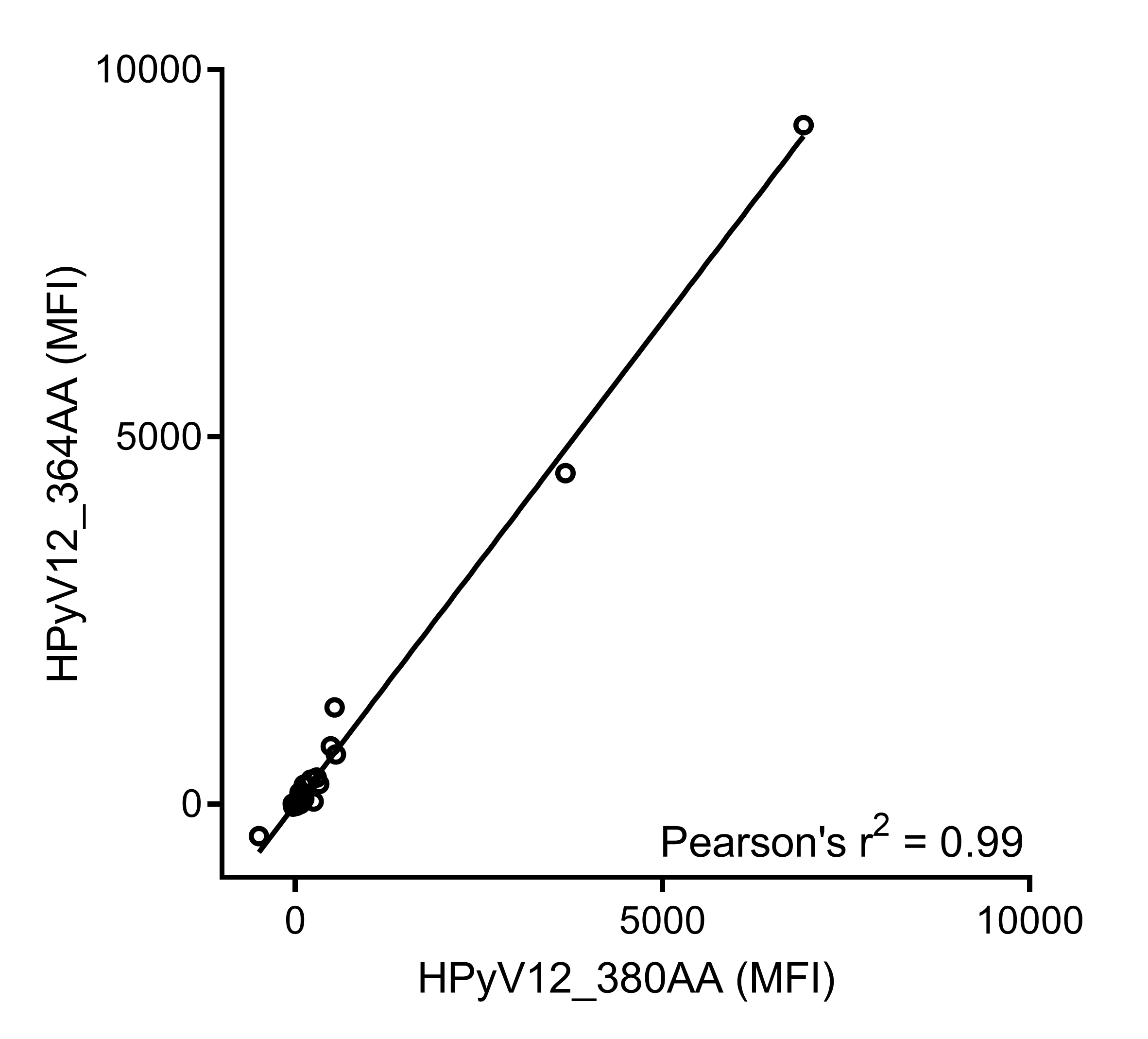

Supplement: S1 Fig — Based on VP1 sequence alignment, the translation initiation site of the HPyV12-VP1 sequence may be located 48 nucleotides (16 amino acids) downstream of the 5’ end of the VP1 open reading frame. In order to compare the antigenicity, both the 380 amino acids long version and the 364 amino acids long version of the HPyV12-VP1 protein were expressed and used to analyse a cohort of kidney transplant recipients (n = 65). The seroreactivity measured with each protein was similar (Pearson R2 = 0.99). (TIF) [file pone.0206273.s001.tif]
